# Supplementary material for: From Glaphene to Glaphynes: A Hybridization of Two-Dimensional Silica Glass and Graphynes
Source: ACS Nano. 2026 Feb 11;20(7):5541–9. doi: 10.1021/acsnano.5c16085 (PMC12947738; doi:10.1021/acsnano.5c16085)
Supplement: Supplementary file 1 [file nn5c16085_si_001.pdf]

Supporting Information: From Glaphene to Glaphynes: A hybridization of 2D silica glass and graphynes

Guilherme S. L. Fabris,[†] Raphael B. de Oliveira,[‡] Marcelo L. Pereira Jr,[*,‡,¶] Robert Vajtai,[‡] Pulickel M. Ajayan,[‡] and Douglas S. Galvão[*,†,§]

†*Applied Physics Department and Center for Computational Engineering & Sciences, State University of Campinas, Campinas, São Paulo 13083970, Brazil.*

‡*Department of Materials Science and NanoEngineering, Rice University, Houston, TX 77005, USA.*

¶*University of Brasília, College of Technology, Department of Electrical Engineering, Brasília, Federal District, Brazil.*

§*Center for Computational Engineering & Sciences (CCES), State University of Campinas, Campinas, São Paulo 13083970, Brazil.*

E-mail: mp200@rice.edu; galvao@ifi.unicamp.br

Ab initio molecular dynamics

Thermal stability was investigated through *ab initio* molecular dynamics (AIMD) simulations performed with DFTB+[S1] using the velocity Verlet integrator in an NPT ensemble at 300 K. A total simulation time of 30 ps with a time step of 1 fs was adopted, and the external pressure was fixed at 0 Pa using a barostat to allow for complete relaxation of the in-plane cell parameters during the thermalization process. Throughout the simulations,

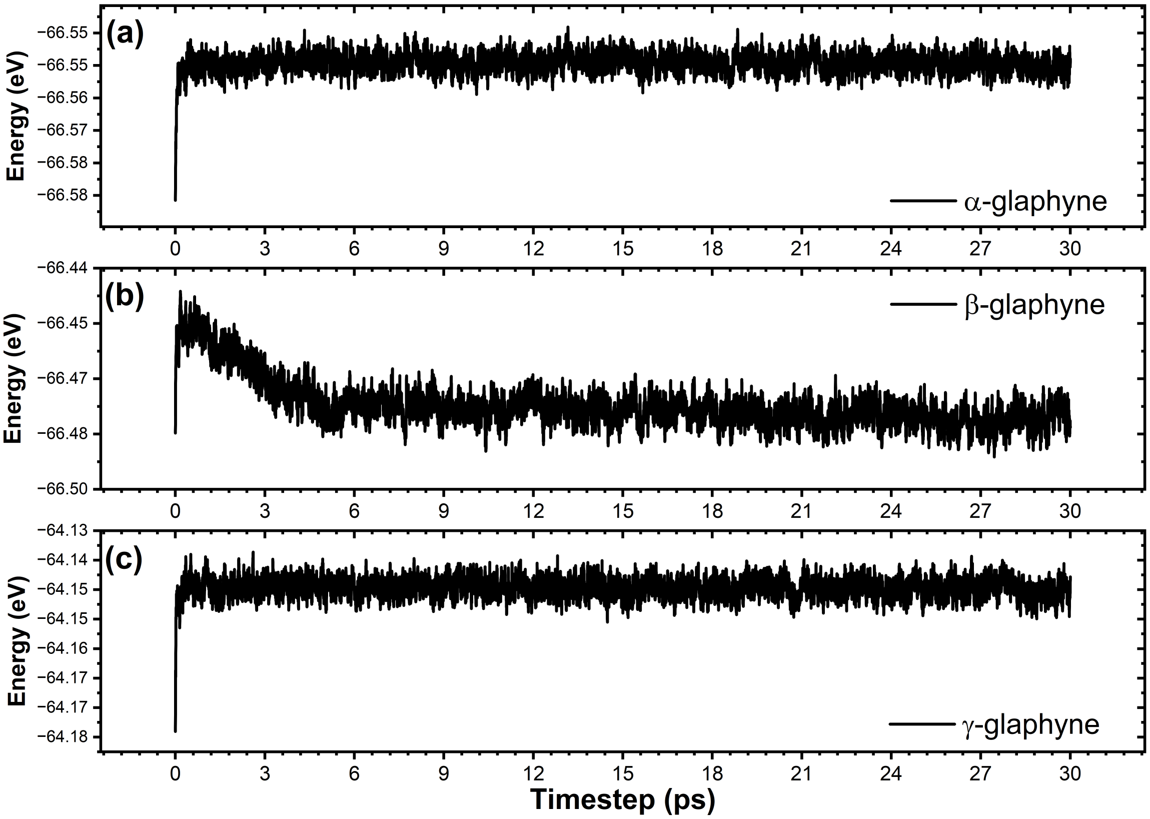

Figure S1: Total energy evolution during AIMD simulations at 300 K for (a) α-glaphyne, (b) β-glaphyne, and (c) γ-glaphyne.

all glaphyne allotropes preserved their structural integrity without reconstruction or bond breaking, indicating robust stability of the carbon skeleton.

Figure S1 shows the total energy evolution for α-, β-, and γ-glaphyne. After an initial equilibration transient, all systems exhibit only small-amplitude fluctuations around a stable mean value, confirming dynamical stability and the absence of thermally activated instabilities within the investigated timescale.

Phonon dispersion calculations

Phonon dispersion relations were computed for β-glaphyne using PHONOPY[S2] based on a 3×3 supercell generated from the fully optimized structure. Finite displacements obtained force constants, and the phonon spectrum was calculated along high-symmetry paths of the

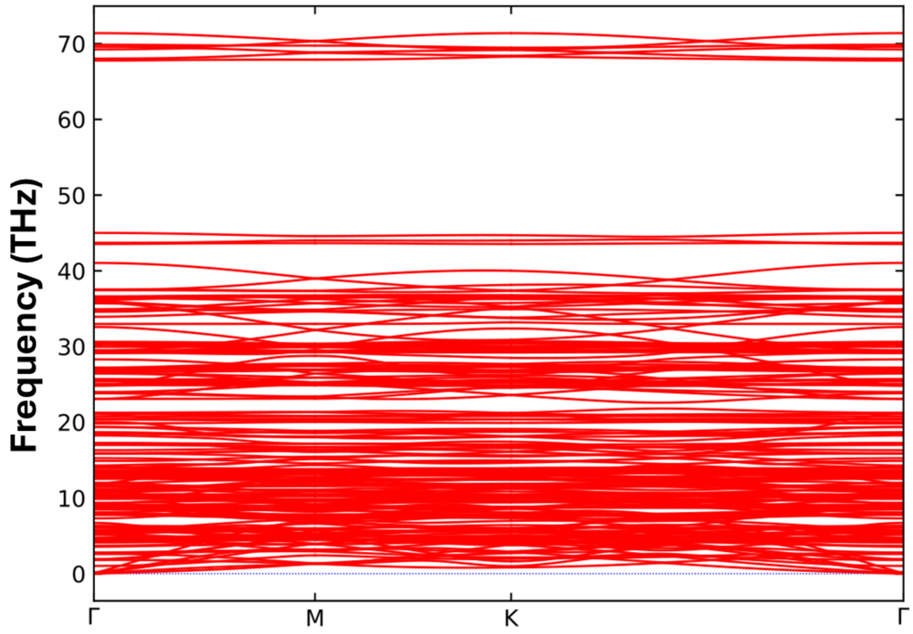

Figure S2: Phonon dispersion for β-glaphyne computed with PHONOPY using a 3×3 supercell.

Brillouin zone. The resulting phonon branches are displayed in Figure S2.

The absence of imaginary modes throughout the Brillouin zone confirms the mechanical dynamical stability of β-glaphyne at 0 K. High-frequency optical modes near 70 THz originate from the acetylenic C≡C units, while the mid-frequency bands reflect the coupling between the hexagonal rings and linear carbon linkages.

Benchmark electronic band structure

Benchmark density functional theory (DFT) calculations were performed for β-glaphyne using the CRYSTAL17[S3] code with PBE, BLYP, HSE06, B3LYP, and B3LYP-D3 functionals. All-electron basis sets were employed, specifically TZVP[S4] for carbon, 86-311G** [S5] for silicon, and 8-411d1[S6] for oxygen. Convergence thresholds for the root mean square of the gradient and nuclear displacements were set to 0.001 and 0.003 a.u., respectively. All stationary points were verified as true minima through Hessian matrix diagonalization and the absence of imaginary vibrational modes.

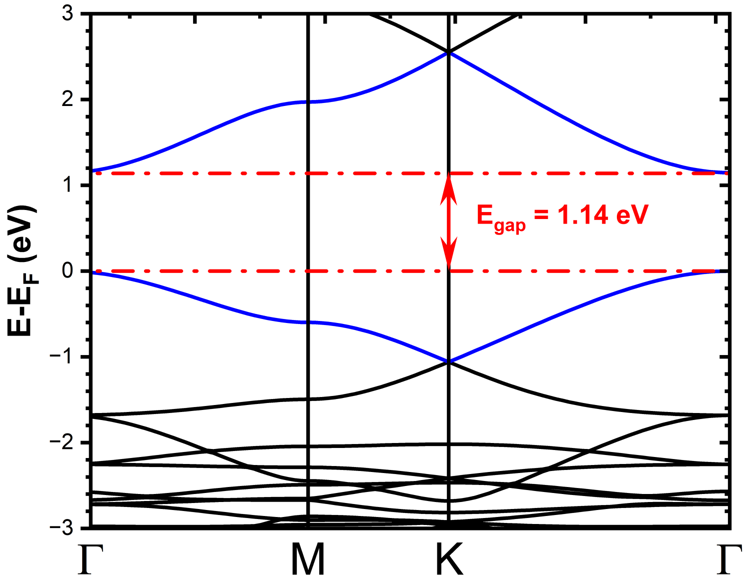

Figure S3: Electronic band structure of β-glaphyne obtained from hybrid-functional DFT calculations.

The electronic structure was evaluated along the conventional high-symmetry Γ–M–K–Γ path of the first Brillouin zone. Figure S3 presents the B3LYP-D3 band structure, showing that β-glaphyne exhibits a direct electronic band gap of approximately 1.14 eV at the K point. The conduction and valence band edges are primarily composed of p_z-derived π states, reflecting the characteristic electronic topology of acetylenic graphyne frameworks.

References

(S1) Hourahine, B.; Aradi, B.; Blum, V.; Bonafé, F.; Buccheri, A.; Camacho, C.; Cevallos, C.; Deshaye, M. Y.; Dumitrică, T.; Dominguez, A.; Ehlert, S.; Elstner, M.; van der Heide, T.; Hermann, J.; Irle, S.; Kranz, J. J.; Köhler, C.; Kowalczyk, T.; Kubař, T.; Lee, I. S.; Lutsker, V.; Maurer, R. J.; Min, S. K.; Mitchell, I.; Negre, C.; Niehaus, T. A.; Niklasson, A. M. N.; Page, A. J.; Pecchia, A.; Penazzi, G.; Persson, M. P.; Řezáč, J.; Sánchez, C. G.; Sternberg, M.; Stöhr, M.; Stuckenberg, F.;

Tkatchenko, A.; z. Yu, V. W.; Frauenheim, T. DFTB+, a software package for efficient approximate density functional theory based atomistic simulations. *The Journal of Chemical Physics* **2020**, *152*, 124101.

(S2) Togo, A. First-principles Phonon Calculations with Phonopy and Phono3py. *Journal of the Physical Society of Japan* **2023**, *92*.

(S3) Dovesi, R.; Erba, A.; Orlando, R.; Zicovich-Wilson, C. M.; Civalleri, B.; Maschio, L.; Rérat, M.; Casassa, S.; Baima, J.; Salustro, S.; Kirtman, B. Quantum-mechanical condensed matter simulations with CRYSTAL. *WIREs Computational Molecular Science* **2018**, *8*.

(S4) Peintinger, M. F.; Oliveira, D. V.; Bredow, T. Consistent Gaussian basis sets of triple-zeta valence with polarization quality for solid-state calculations. *Journal of Computational Chemistry* **2012**, *34*, 451–459.

(S5) Pascale, F.; Zicovich-Wilson, C. M.; Orlando, R.; Roetti, C.; Ugliengo, P.; Dovesi, R. Vibration Frequencies of Mg3Al2Si3O12 Pyrope. An ab Initio Study with the CRYSTAL Code. *The Journal of Physical Chemistry B* **2005**, *109*, 6146–6152.

(S6) Bredow, T.; Jug, K.; Evarestov, R. A. Electronic and magnetic structure of ScMnO3. *physica status solidi (b)* **2006**, *243*.
